# Diversity of medium and large mammals in the Loka Abaya National Park, southern Ethiopia

**DOI:** 10.1002/ece3.6649

**Published:** 2020-08-28

**Authors:** Guta Diriba, Sintaheyu Tamene, Girma Mengesha, Addisu Asefa

**Affiliations:** ^1^ Department of Wildlife & Eco‐tourism Wondo Genet College of Forestry & Natural Resources Shashamanne Ethiopia; ^2^ Ethiopian Wildlife Conservation Authority Project Addis Ababa Ethiopia

**Keywords:** distribution, diversity, Loka Abaya National Park, mammals, relative abundance, species richness

## Abstract

We evaluated the richness, diversity, and composition of the medium and large mammal community in the Loka Abaya National Park (LANP), southern Ethiopia, and how these parameters differ among four habitat types: wooded grassland, riverine forest, hilly scrubland and wetland, and between seasons. We recorded a total of 2,573 individual animals of 28 medium and large mammal species in the park. This included three globally threatened species: the endangered African wild dog (*Lycaon pictus*), the vulnerable Leopard (*Panthera pardus*), and Hippopotamus (*Hippopothamus amphibius*). Season had little effect on species richness, diversity, and composition both across and within habitat types. However, species richness across seasons was significantly different among the four habitat types, in the declining order of the following: wooded grassland > riverine forest > hilly scrubland > wetland. The strongest similarity in species composition, both across and within seasons, was found between wooded grassland and riverine forest. In terms of relative abundance, mammal assemblage of the wooded grassland and wetland habitats had more evenly distributed number of species with different relative abundance categories. Overall, Anubis Baboon (*Papio anubis*), Grivet Monkey (*Chlorocebus aethiops*), and Greater Kudu (*Tragelephus strepsiceros*) were the three most abundant species across habitat types. In conclusion, findings of our study reveal that LANP plays an important role in Ethiopia's mammal conservation. Our findings will serve as baseline information for managers of the park to make effective conservation decisions and as a baseline for researchers wishing to conduct related ecological studies.

## Graphical abstract

We investigated and determined medium and large mammalian diversity and identified habitat use and seasonal variation. We were able to record endangered and vulnerable mammals in the Loke Abaya National park.

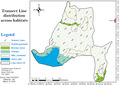

## 1 INTRODUCTION

Creation of protected areas, such as National Parks, has globally been considered as the principal strategy for biodiversity conservation, climate change mitigation and adaptation (Bernard, Penna, & ArauÂjo, 2014; Locke & Dearden, 2005). As a result, the number and size of protected areas have been showing increasing trends worldwide (Bernard et al., 2014). Despite such heavy reliance on protected areas as conservation strategy and the increasing trends in their number and coverage, many protected areas are in danger of not achieving the specific conservation goals for which they were originally created (Bernard et al., 2014; Struhsaker, Struhsaker, & Siex, 2005). Increased anthropogenic threats, poor management systems, and limited finances are the major challenges to achieve conservation goals of protected areas (Bernard et al., 2014; Bruner, Gullison, Rice, & da Fonseca, 2001; Millennium Ecosystem Assessment [MA], 2007). Currently, the loss of biodiversity has become a major global environmental concern because it does not entail only loss of species, but also entails disruption of ecosystem processes and loss of the ecosystem services and benefits they provide to human beings (MA, 2007). Unless effective conservation measures are in place, the future existence of biodiversity in such protected areas, particularly those of developing tropical countries like Ethiopia, is therefore under question. The first important information needed to develop effective conservation strategies of such protected areas is having basic information on the biodiversity they contain, including species checklists of fauna and flora and the distribution and habitat use of wildlife species. This information aids decision makers, and conservation agencies understand the conservation values of protected areas, prioritize areas accordingly, and clearly define and implement effective conservation actions (Bernard et al., 2014; Thomas & Middleton, 2003). Surprisingly, such basic information is not available for most protected areas, especially in African countries such as Ethiopia.

Ethiopia is among the African countries hosting a high diversity and endemism of plant and animal species (Yalden & Largen, 1992; Yalden, Largen, & Kock, 1986). The country is known to contain, among others, 6,500 species of plants (with 600 endemic species), 320 species of mammals (55 are endemics), and 918 species of birds (18 endemics) (Amare, 2015; Gonfa, Gadisa, & Habtamu, 2015). The primary factor responsible for such diversity and endemism is the existence of diverse habitats, ecosystems and other environmental variables that created favorable conditions for the evolution and persistence of species (Hillman, 1993a). Of the key measures taken by successive Ethiopian governments to conserve the declining populations of species, have been establishing protected areas such as National Parks, Wildlife Sanctuaries, and Wildlife Reserves. Currently, the country has established 73 protected areas of different categories, including 25 National Parks (Tessema, Wakjira, & Asefa, 2019). However, like the case of many African countries, several key wildlife species have shown declining trends both in population sizes and ranges of distribution due to habitat loss and fragmentation and hunting (Abune, 2000; Yalden & Largen, 1992). Furthermore, many of these protected areas also lack basic ecological information, without which it is hardly possible to evaluate trends of the animal populations, management effectiveness, and impractical to practice effective conservation activities. Therefore, it is a matter of urgency to inventorize mammal species in particular across protected areas where such information is lacking.

Studies of mammals are necessary because they are an important ecological constituent of various ecosystems and thus have significant ecosystem functions and provide vital ecosystem services to human beings (Geleta & Bekele, 2016). For example, they serve as food sources and raw materials for production of basic human needs; regulate plant diversity, structure, and potential pest species through herbivory; plant dispersion through seeds consumption; and predators control other animal populations through predation (Carvalho, Oliveira, & Pires, 2014; Cortés–Marcial, 2014). Many mammal species also act as a flagship for public awareness on the conservation values of biodiversity, and as umbrella species because of their large area home range requirements which contribute to the conservation of other species (Sillero‐Zubiri et al., 2011). Despite this, numerous recent anthropogenic factors have promoted habitat loss and fragmentation, leading to the decline and losses of global mammalian biodiversity (Heinze et al., 2011; IUCN, 2020; Struhsaker et al., 2005). The ecological relevance of mammals, shortage of ecological data, and increased human threats make the matter very essential and necessary to evaluate their current conservation status (Atnafu & Yihune, 2018). Hence, surveys of large and medium mammalian diversity of a particular ecosystem are the first step for conservation action and provide information to establish appropriate conservation strategies (Bernardo & Melo, 2013). The understanding of how mammalian species persist in different locally available habitats may also indicate the requirements of species and might contribute to their conservation (Bernardo & Melo, 2013). This is particularly pertinent because, in addition to anthropogenic activities, the presence of a species and its distribution among available habitats in a given area are influenced by several ecological factors, such as habitat quality and suitability (Fetene, Mengesha, & Bekele, 2011; Heinze et al., 2011; Mamo, Bekele, & Mengesha, 2012), presence of other species (e.g., superior competitors and/or predators) (Mamo, Asefa, & Mengesha, 2015). Ultimately, the individual and interactive effects of such anthropogenic and ecological factors shape the patterns of species richness and diversity, be it at local level (e.g., within a habitat type) or landscape level (Asefa, Mengesha, Sori, & Mamo, 2019; Mamo et al., 2015). Thus, having information on the spatial and temporal patterns of diversity of biological taxa is important to understand underlying factors and to address them, particularly when such changes are primarily driven by direct or indirect human actions (Illius & O'Connor, 2000; Mamo et al., 2015; Morrison, Marcot, & Mannan, 1998; Stankowich, 2008).

Loka Abaya National Park (LANP), southern Ethiopia, was established in 2009 to conserve key wildlife species such as the Lesser Kudu (*Tragelaghus imberbis*), Defassa Waterbuck (*Kobus defassa*), Common Bushbuck (*Tragelaghus scriptus*), Lion (*Panthera leo*), Leopard (*Pantera pardus*), and African wild dog (*Lycaon pictus*) (SNNPRS‐C SZBCT, 2009). Furthermore, Lake Abaya, the largest lake in the Ethiopian Rift Valley system, is located inside the park. In addition to regulating the drainage of a number of water basins into the lake, the LANP also plays a crucial role in regulating ecosystem processes and functions of the lake. Despite this, the park has been under human interference (e.g., poaching, cultivation, uncontrolled fire, fire wood collection, and logging for charcoal production) and livestock pressures (Demeke, Tamene, Kifle, & Mengesha, 2019; SZBCT, 2009). Such human‐induced actions can adversely affect wildlife of the park. While urgent management actions are needed to abate these threats and mitigate the actual and potential impacts on biodiversity, it is also important to have an understanding of the status of prominent biological components such as mammals. Such understanding would assist managers of the park determine magnitude of the impacts and take more effective, informed management decisions. However, published basic ecological information on biodiversity of the Park, including mammals, has been lacking. Therefore, this study was conducted to provide basic information on medium and large (i.e., species with a body mass of over 5 kg; Stephens, d'Sa, Sillero‐Zurbri, & Leader‐Williams, 2001; Geleta & Bekele, 2016) that would aid decision making and promote future research. The specific objectives were to determine the species diversity (richness, evenness, Shannon diversity, and species composition of mammals of the park), and to assess how this diversity may across spatial (habitat types) and temporal (seasonal) scale.

## 2 MATERIALS AND METHODS

### 2.1 Study area

**FIGURE 1:**
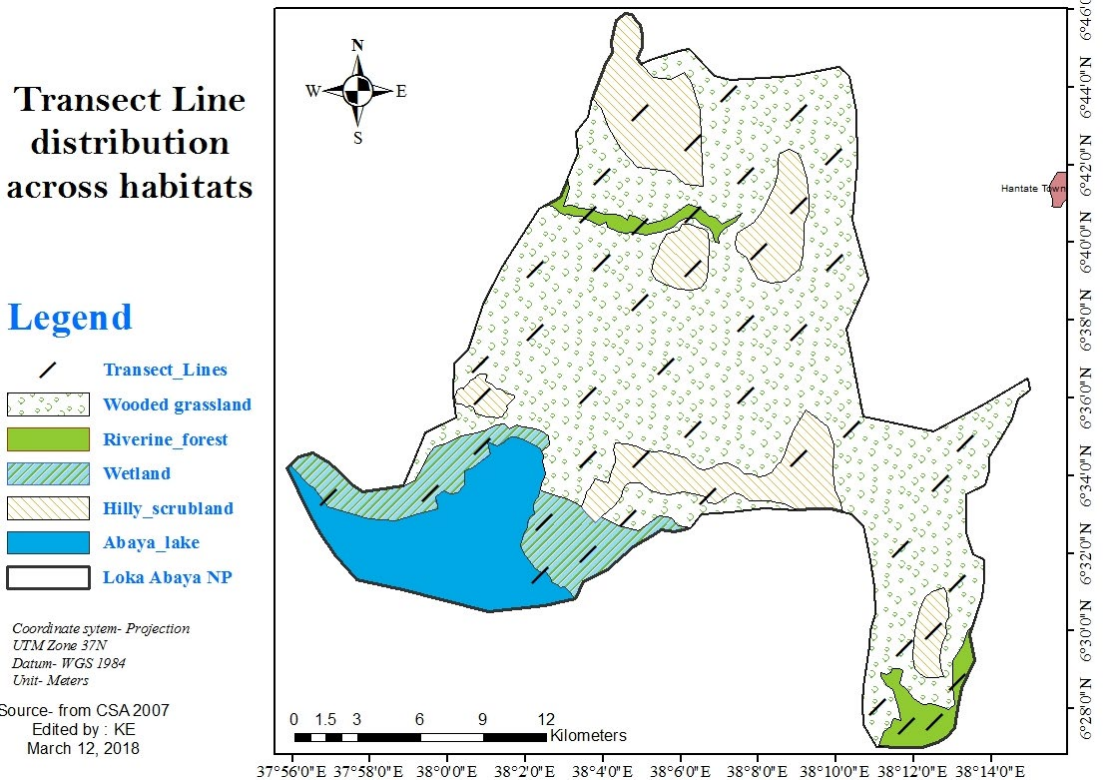
Location of study area and sampled area where transect line distributed among different habitats in the study area. (CSA, 2007; Landsat ETM+ scene L171168055‐0552051203. ETMGLS, 2015)

Loka Abaya National Park (6°27′00″–6° 46′00″ N; and 37°55′00″–38°15′00″ E) is located in Southern Nations Nationalities and Peoples Regional State (SNNPRS) of Ethiopia, southwest of Hawassa (capital of the region) and Addis Ababa (capital of the country) at distance of 70 km and 340 km, respectively (SZBCT, 2009). Specifically, it is situated in Loka Abaya *Woreda* (District) of the Sidama zone administration (Figure 1; SZBCT, 2009). It was established in 2009 to conserve wildlife and ecosystem of the area. The LANP has an area of 500 km^2^ of which 448 km^2^ is terrestrial while 52 km^2^ is water body (covered by the Lake Abaya) (Figure 1). The altitudinal range of the park is 1,000–1,800 m a.s.l. The prominent topographic features of the LANP are characterized by highly heterogeneous and hilly terrain. Large proportion of the study area is highly undulating and rolling interspersed with different valley floors, purely drained bottom land and punctuated by different hills. The sample distribution across the areas is shown in FIgure 1 below.

The study area has bimodal rain fall: July to September (heavy rains) and February to April (light rains), the remaining months of the year are fairly dry. The mean annual rainfall and temperature in the area are 1,001–1,400 mm and 17.6–25°C, respectively. Based on vegetation structure and composition, LANP has four broad habitat types (Demeke et al., 2019): wooded grassland (area = 286 km^2^), hilly scrubland (area = 80 km^2^), riverine forest (area = 52 km^2^), and wetland (area = 30 km^2^) (SZBCT, 2009; Figure 1). The dominant plant species in the wooded grassland and hilly scrubland are various Acacia species such as *Acacia drepanolobium, A. melifera, A. seyal, A. tortolies, A. senegal, A. albida,* and *A. nilotica*. Other species mostly in wooded grassland include *Aloe vera, Euphorbia tirucalli, Dodinia viscose,* and *Balanities aegyptica*. The riverine forest is dominated by tree species like *Ficus sur, F. vasta, Petroletum stelatium,* and *Temarindus indica*. The wetland habitat is covered by grass and sedge species (Demeke et al., 2019; SZBCT, 2009).

### 2.2 Data collection

Fixed‐width line transect sampling method was used to collect mammalian data (Sutherland, 2006). Based on satellite images and preliminary survey, the study area was first stratified into the four habitat types described above: wooded grassland, hilly scrubland, riverine forest, and wetland habitat. This was delineated on a top map of the area, and transects were then established systematically in representative (homogenous vegetation) areas of each habitat type. The distance between adjacent transects and from habitat edges to a transect was limited to a minimum of 1 km, to avoid double counting and to avoid edge effects, respectively (Krebs, 1989). A total of 48 line transects were established across the four major habitat types. Number of transects varied among habitats depending on their size: 26 in the wooded grassland, 10 in the hilly scrubland, and 6 each in the riverine forest and wetland habitats. The length of each transect line was 5 km and a fixed‐sighting distance of 200 m on both sides of transects was used in the wooded grassland, hilly scrubland, and wetland habitats. Sighting distance in the riverine forest was fixed to 100 m because of greater vegetation thickness that obscure accurate observation and identification of mammals beyond 100 m distance from the transect lines. The starting and ending points of each transect were fed into a Garmin GPS unit and used for navigation during data collection.

Mammal surveys were carried out from August to October 2017 during the wet season and January to March 2018 during the dry season. Data collection was conducted while walking quietly and gently along each transect and recording animal observations. Data recorded whenever an individual animal or group of animals sighted were as follows: date, time, habitat type, species name, individual number of each species, and GPS location (Girma, Mamo, & Ersado, 2012; Gonfa et al., 2015; Mengesha & Bekele, 2008). Animal counting was made by naked eye and using 7 × 50 mm and 8 × 40 mm Canon binoculars. Whenever deemed necessary, Kingdon's (2003) field guide book was used for identification of mammals. In addition to direct observation, indirect evidences like fecal droppings, feed marks, foot print, dens, territorial markings, spine, call, and other evidences were also recorded (Sutherland, 2006). However, species identified from data obtained from indirect evidence were subsets of the species observed during the standard survey, thus were not used for data analysis.

Surveys were carried out when most animals are thought to be more active; early in the morning between 6:00 and 10:00 hr and late in the afternoon between 15:00 and 18:00 hr (Sutherland, 2006). Each transect line was surveyed twice on a given day during each season; thus, all transects were visited four times in the course of the study period. Data from the two replicate surveys each season were pooled together for each transect and used for analysis (Girma et al., 2012; Mengesha & Bekele, 2008).

### 2.3 Data analysis

We computed species diversity in four ways: species richness (number of species found at a particular area during a given time period), species diversity (combination of species richness and evenness), species composition (the similarity in species composition between two treatment categories, which in the present case was habitat types, or seasons) (Magurran, 2004; Mamo et al., 2015), and species relative abundance (a measure of evenness of abundance distribution).

We used an individual‐based rarefaction and extrapolation methods to estimate species richness in EstimateS 9.1 (Colwell, 2013). The summed abundance of the number of individuals of each species recorded along each transect for each habitat type in each season was used as the input for the individual‐based richness computation. During richness computation, we used 100 sample order randomizations (i.e., the order in which individual species' abundances added to the analysis within each study site or season) without replacement. We estimated species richness using the Chao 1 estimator (Colwell, 2013). We assessed sampling completeness by comparing the observed and Chao 1 estimated species richness (Colwell, 2013). We did all these richness analyses for the study area (habitats combined) both across and within season, and for each study habitat both across and within season. We assessed sampling completeness by comparing the observed S(obs) and estimated S(est) (based on Chao 1) species richness values (Colwell, 2013). To compare estimated (based on rarefaction and extrapolation) species richness among treatments (habitat types or seasons), we computed estimated species richness S(est) with 95% confidence intervals (CI). Following various authors (e.g., Clarke & Gorley, 2006; Asefa, Davies, McKechnie, Kinahan, & van Rensburg, 2017; Colwell et al., 2012), we used nonoverlapping 95% CIs of S(est) (based on extrapolated) at the reference sample size (sample size of the habitat with the largest sample size) as a conservative criterion of statistical difference (at alpha = 0.05) in species richness between habitat types. Species diversity was computed using the Shannon diversity (H) index in EstimateS.

We examined pattern of evenness of species abundance in two approaches. First, we calculated the relationship of abundance of each mammal species between treatments (habitat types and seasons) using Spearman's rank correlation (*r_s_*) in SPSS version 20 (IBM, 2011). In the second approach, we calculated the relative abundance of each species in each treatment (e.g., number of individuals of a species recorded in the riverine forest during dry season divided by the total number of animals recorded in the riverine forest during the dry season, and multiplied by 100) and subjectively grouped species into four crude relative abundance categories, as: abundant (species with relative abundance greater than the third quartile), common (species with relative abundances greater than the second quartile or equal to the third quartile), uncommon (species with relative abundance greater than the first quartile or equal to the second quartile), and rare (species with relative abundance less than or equal to the first quartile) (Hillman, 1993b; Negeri, Gadisa, & Habtamu, 2015). Then, variations in number of species belonging to each relative abundance category among habitats were tested using chi‐square test in SPSS ver 20 (IBM, 2011).

To examine species composition of habitat types, we conducted Bray–Curtis similarity analysis between each pair of habitats, based on square‐root transformed data, in Primer version 6 application (Clarke & Gorley, 2006). We also undertook a nonmetric multidimensional scaling analysis to ordinate the assemblage of each habitat in each season.

## 3 RESULTS

### 3.1 Species composition and richness

A total of 2,573 individual animals of 28 medium and large mammal species, belonging to 14 families and 7 orders, were recorded in the park during the study period (Appendix S1). Of these, 1,214 (47%) and 1,359 (53%) animals were observed during the wet and the dry seasons, respectively (Table 1). Carnivora was the first and the second most abundant order in terms of number of families (5 families) and species (10 species), respectively. Order Artiodactyla was the second and the first most abundant in terms of number of families (3 families) and species (11 species), respectively. Whereas, four mammalian orders were represented by a single species. At family level, Bovidae (8 species) and Canidae (4 species) were the dominant families, but eight families were represented each by a single species (Appendix S1).

**TABLE 1:** Number of individuals and observed S(obs) and estimated Chao 1 species richness of mammals in the four habitat types of the Loka Abaya National Park

Habitat/season	Individuals	S(obs) mean (95% CI)	Chao 1 mean (95% CI)
Overall	2,573	28	28 (0.28)
Wooded grassland	1,585	27	27 (1.25)
Hilly scrubland	313	19 (1.37)	19 (1.12)
Riverine forest	624	23 (0.97)	23 (0.42)
Wetland	51	7 (0.47)	7 (1.43)
Wet season	1,214	28	28 (0.94)
Dry season	1,359	28	28 (1.01)

Note

Species richness values are mean and their 95% CIs (based on 100 times sample randomizations) and those mean values without CI are those with CI equals the means due to *SD* getting closer to zero at the maximum sample accumulation curve.

**FIGURE 2:**
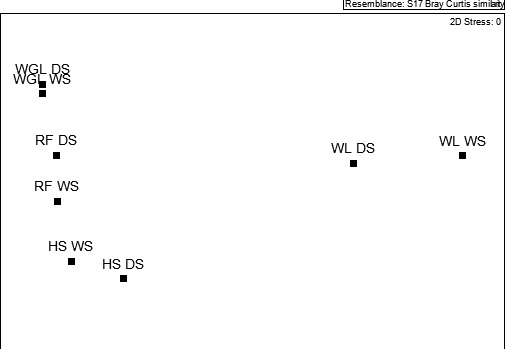
Nonmetric multidimensional scaling of habitat types in each season

Based on the Bray–Curtis similarity (*S_i_*) analysis, species composition of mammalian assemblages of the study area showed high similarity (94%) between the wet and dry seasons. The strongest similarity between habitats in species composition, both across and within seasons, was between the wooded grassland and riverine forest (*S_i_* = 74%), followed by between the latter habitat and hilly scrubland (*D_s_* = 62%). However, the similarity between the wetland habitat and each of the other three habitat types was weak (ranged between 16%–26%) (Table 2a,b). Similarity in species composition within each habitat between seasons was strongest for the wooded grassland (*D_s_* = 94) and lowest for the wetland (57) habitat types (Table 2a). These results suggest the weaker effect of season on species composition of the study area compared with habitat type. This weak seasonal effect was also clearly demonstrated that in the wet and dry seasons assemblages of each habitat type were ordinated more closely to each other on the multidimensional space (Figure 2).

**TABLE 2:** Bray–Curtis similarity index: (a) between each pair of habitat types (entries above diagonal top‐right) and between dry and wet seasons within each habitat type (entries along the diagonal), and (b) between each pair of habitat types during the wet season (entries below diagonal in the bottom‐left) and during the dry season (entries above diagonal in the top‐right)

Habitat	Wooded grassland	Riverine forest	Hilly scrubland	Wetland
(a) Across and between season				
Wooded grassland	94	74	55	16
Riverine forest		85	62	23
Hilly scrub			75	26
Wetland				57
(b) Within season				
Wooded grassland		79	43	19
Riverine forest	65		48	26
Hilly scrub	59	59		36
Wetland	7	10	6	

**FIGURE 3:**
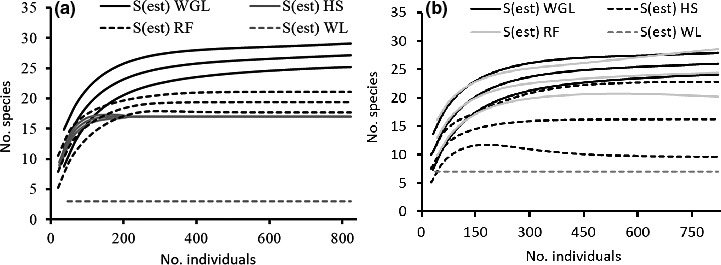
Species richness of the four habitat types during wet season (a) and during dry season (b). Habitats: WGL = wooded grassland; HS = hilly scrubland; RF = riverine forest; WL = wetland

**FIGURE 4:**
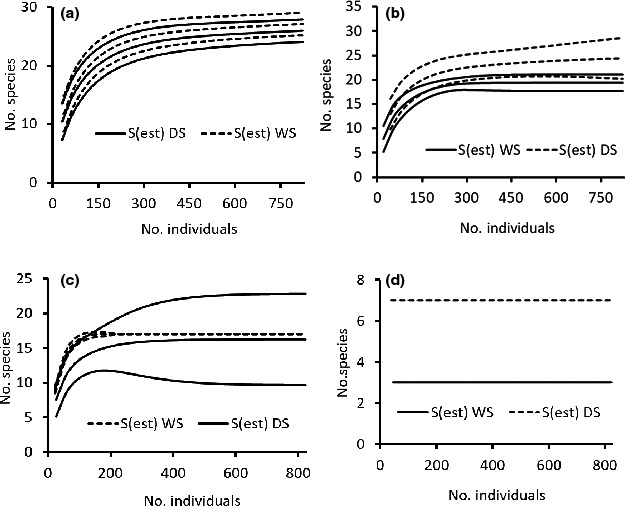
Seasonal (WS = wet season, DS = dry season) species richness within each habitat type (a) a) wooded grassland, riverine forest (b), hilly scrub (c), and wetland (d)

Comparison of observed species richness of the study area and each treatment (habitat type or season) with the estimated richness (based on Chao 1) showed that sample completeness was greater than 95% (see Table 1). Further, results of observed and interpolated richness were qualitatively similar. Thus, results of extrapolation were presented and discussed throughout the article. At landscape level (across habitats), Chao 1 estimated species richness was 28 (95% CI: 27.72–28.28), indicating that almost all the species expected to be found in the area were recorded. Estimated species richness across habitat types was not significantly different between the dry and wet seasons (Table 1). At habitat level, mammal species richness across season was significantly different among the four habitat types, in declining order of: wooded grassland > riverine forest > hilly scrubland > wetland (Table 1). Similar results were found when species richness of habitats was compared within each season, except the nonsignificant difference found during the dry season between wooded grassland and riverine forest, and between riverine forest and hilly scrub habitat types (Figure 3a,b). Seasonal significant difference in species richness within a habitat type was revealed only for the wetland habitat type, which was higher during the dry season (Figure 4a‐d).

### 3.2 Species diversity and relative abundance

Regardless of habitat type, Shannon diversity index was similar during the dry and the wet seasons (Table 3). Irrespective of season, Shannon diversity index was highest in the wooded grassland, followed by riverine forest, hilly scrub, and wetland habitat types (Table 3). Considering within habitat type, the wooded grassland and the hilly scrub habitat types had higher diversity during the wet season compared with the dry season, and *vice versa* for the riverine forest and wetland habitats (Table 3).

**TABLE 3:** Shannon heterogeneity index of each habitat type across season and within season (season combined) and each season across habitats

Habitat/season	Season combined	Wet season	Dry season
All habitats	2.36	2.34	2.36
Wooded grassland	2.31	2.33	2.28
Riverine forest	2.27	2.10	2.36
Hilly scrubland	1.98	2.08	1.66
Wetland	1.69	1.07	1.77

**FIGURE 5:**
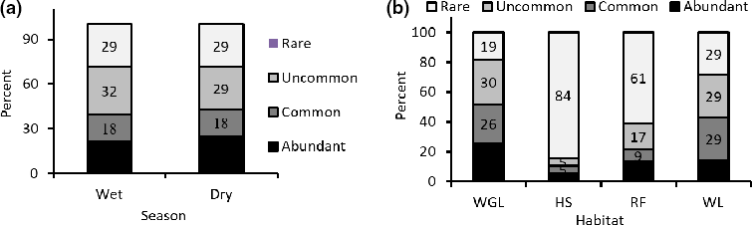
Percentage contribution of number of mammal species with the different relative abundance categories in the wet and dry seasons (a) and in each of the four habitat types (b). Habitat: WGL = wooded grassland; HS = hilly scrubland; RF = riverine forest; WL = wetland; WS = wet season; DS = dry season. (Values for each category are given on the graph as data label.)

There was a strong correlation in rank‐abundance of mammal species across habitats (combined habitats) between the dry and wet seasons (*r_s_* = 0.92). Without considering season, the correlation of rank‐abundance of mammal species between habitat types showed stronger relationship between the wooded grassland habitat and the hilly scrubland (*r_s_* = 0.72), followed by between the former and the riverine forest (*r_s_* = 0.54) (Table 4). Within habitat type, the wooded grassland habitat also had the highest correlation between the rank‐abundances of the wet and dry seasons assemblages (*r_s_* = 0.91, followed by the wetland habitat (*r_s_* = 0.77) (Table 4). Species' abundance evenness (as measured by the number of species categorized in the four abundance categories) was similar between dry and wet seasons (χ^2^ = 0.99, *df* = 3, *p* = .136; Figure 5a), but significantly varied among habitat types (χ^2^ = 22.73, *df* = 9, *p* < .01; Figure 5b). As shown on Figure 5b, wooded grassland and wetland habitats had more evenly distributed species' relative abundance categories, while most species in hilly scrubland (84% of the total species) and in the riverine forest (61%) did fall in the “Rare” relative abundance category. Overall, *Papio anubis, Chlorocebus aethiops,* and *Tragelephus strepsiceros* were the three most abundant species (with relative abundance falling in the fourth quartile range) in both wooded grassland and the riverine habitat types. *P. anubis*, *T. strepsiceros,* and *Phacochoerus africanus* were also the three most abundant species in the hilly scrub habitat. Mammals of the wetland habitat, however, were dominated by *Kobes ellipsiprymnus, P. africanus,* and *Hippopothamus amphibius* (Appendix S1). All these species were also the most abundant across habitat types.

**TABLE 4:** Rank correlation of abundance of mammal species between habitat types (entries below diagonal bottom‐left) and between dry and wet seasons within each habitat type (entries along the diagonal)

Habitats	Wooded grassland	Riverine forest	Hilly scrub	Wetland
Wooded grassland	0.91			
Riverine forest	0.54	0.34		
Hilly scrubland	0.72	0.21	0.52	
Wetland	−0.07	−0.01	0.07	0.77

## 4 DISCUSSION

Our results indicate 28 species of medium and large mammal species to occur in the LANP including three globally threatened species: the endangered African wild dog (*L. pictus*) and the “vulnerable” Leopard (*P. pardus*) and Hippopotamus (*H. amphibius*) (IUCN, 2020). All these species are also nationally legally protected (FDRE, 2009). Even large predators, such as Hyena, Hunting dog, and *P. pardus*, that are killed in retaliation for attacks on domestic animals still are persisting in the park throughout the year. The presence of these conservation concern species demonstrates the effectiveness of wildlife conservation in the protected areas (Bernard et al., 2014; Bruner et al., 2001). It is a common understanding that the number of species detected at a given study area during a particular survey time is a function of sampling effort, more species could be recorded when additional sample units are surveyed (Colwell et al., 2012). However, this may not be the case in our study because the rarefaction, extrapolation, and estimated (Chao 1) richness curves formed plateau and observed species was similar to expected richness in all treatments, all suggesting sampling completeness (i.e., all the species present in the study are detected). Our results also show that season has little influence on the species richness, diversity, and composition, but habitat type has significant influence. All the species encountered in the riverine forest, hilly scrubland, and wetland (except *H. amorphous*) habitats are subsets of the species recorded in the wooded grassland habitat (Appendix S1 & Table 1).

Overall, the number of species recorded in the park is comparable with reports of medium and large mammals from other protected areas of Ethiopia. For example, 30 species have been reported from Maize National Park (Yimer & Yirga, 2013); 28 species from the Dati Wolel National Park (Gonfa et al., 2015); 23 species from Borena Saint National Park (Chane & Yirga, 2014); and 23 species from Baroye Controlled Hunting area (Negeri et al., 2015). Findings of the present study therefore highlight that LANP has a valuable importance for the conservation of Ethiopia's mammal species. The occurrence of *L*. *pictus* in the area throughout the year is particularly interesting, because in addition to the species' low global population, their long‐distance mobility (a territory size of 350–950 km^2^), lack of sufficient diet and disturbance from many communities across their range are thought to decrease their encounter rate (Mills & Gorman, 1997). Therefore, record of the species in the study area throughout the year may indicate the presence of resident population in the area. Although ecological studies of other animal groups are scanty, a recent study by Demeke et al. (2019) has highlighted the importance of LANP for preservation of birds. However, the national park is being constantly and consistently threatened by such human activities as hunting, and cattle grazing and crop cultivation (SNNPRS‐CTB, 2009; Demeke et al., 2019). Thus, appropriate conservation measures should be in place to mitigate the impacts of these threats to wildlife populations and their habitats.

The equal number of species recorded in the study area during the wet and dry seasons could be explained by a number of factors, such as the area's high resilience to seasonal fluctuations (Morrison et al., 1998), lack of suitable habitats in the nearby (Illius & O'Connor, 2000), and/or lack of connectivity and corridor to move to similar areas if present (Alvarenga et al., 2018). Future researches should focus on examining these factors. Nonetheless, the number of individuals recorded during the dry season was higher by a factor 12% than the number of animals recorded during the wet season (Table 1). This seasonal abundance difference contradicts the more expected trend of higher abundance during the wet season, resource availability following the rainfall is expected be higher. The probable reason for this higher number of individuals found during the dry season could be attributed to two main factors: seasonal variations in the level of human disturbances and in complexity of vegetation structure. Human and livestock encroachment into the park is higher during the wet season as the surrounding areas are covered by crops during this season, which causes shortage of grazing land (Worku & Datiko, 2018). Such disturbances affect mammals through various mechanisms, such as leading animals to hide or move to other sites. Thus, in turn, can reduce the probabilities of animals being sighted (Dinakaran & Anbalagan, 2007; Hassani, Asghari, Frid, & Nurberdief, 2008; Stankowich, 2008). In support of this hypothesis, most of the common species of the park, such as *T. strepsiceros, Sylvicapra grimmia, P. anubis, P. africanus,* and *Lepus fagani,* were recorded relatively in lower abundances during the wet season compared with the dry season, which could be attributed to this supposed effect of encroachment on the probability of observing them during field surveys. However, in addition to disturbance, regeneration of woody vegetation and growth of herbaceous and ground vegetation during the wet season might have provided thick cover for the animals, making sighting of the animals difficult (Girma et al., 2012).

The wooded grassland habitat is characterized by greater species richness and Shannon diversity index, both across and within season, and vice versa for wetland habitat. Furthermore, all the mammal species (except *H. amphibius*, which is restricted to the wetland habitat) from the other three habitats were subsets of the species recorded in the wooded grassland. Given the large size of the wooded grassland habitat compared with the others (see Figure 1), these results are unsurprising and agree with the well‐established area–species relationships; which states that habitats with greater area tend to contain higher number of species compared with habitats with smaller area (Bantihun & Bekele, 2015; Girma et al., 2012; Mekonnen, Yaba, Bekele, & Malcolm, 2011). Habitats with large areas usually have diverse microhabitats and more heterogeneous vegetation structure which provide resources (e.g., food and covering space) for species with different feeding and microhabitat requirements (Bantihun & Bekele, 2015; Girma et al., 2012). Specifically, presence of large number of herbivore species guild found in the wooded grassland, as a result of higher habitat quality, might have also attracted a high number of carnivore species and resulting to increased diversity (Alvarenga et al., 2018). As demonstrated in the results (see Appendix S1), most of the carnivore species found in the park were either only recorded in the wooded grassland or had maximum abundance in that habitat type. However, our current understandings of prey–predator relationships and about the use of different habitats by mammals is limited. Further focused studies are needed to improve this understanding and for effective management planning.

Our results also show that mammal species of the study area composed of only one species, *Phacochoerus africanus*, appears to occur across all habitat types, 13 (46%) species in three habitat types, and one habitat specialist species (i.e., *H. amphibius*, which is restricted to the wetland habitat) species (Appendix S1). Consequently, species such as *P. anubis, C. aethiops*, *Colobus geureza*, *T. strepsiceros*, *S. grimmia,* and *P. africanus* were the most abundant species (with relative abundance falling above the third quartile) in the study area or at least in two habitat types. Mammal assemblage of the wetland habitat, however, in addition to *P. africanus,* was dominated by *K. ellipsiprymnus* and *H. amphibius* (Appendix S1). This species' relative abundance trend was also reflected in results of the similarity analysis which highlighted a weaker resemblance of the wetland habitat mammal assemblage with the other three habitat types (see Table 3). This indicates that, despite hosting the lowest number of species, the wetland habitat supports species that are unique to that habitat type, specifically the vulnerable *H. amphibius*. Thus, the wetland habitat plays a complementary role in increasing mammal diversity of the park; as such the low number of species found in the wetland habitat does not imply that this habitat should be neglected during conservation planning. The wooded grassland habitat also exhibited lowest seasonal variations in diversity (Shannon diversity and relative abundance distribution), and conversely highest similarity in species composition compared to within each of the other habitat types. These findings may suggest that the wooded grassland habitat, as described above, is resilient to seasonal resource fluctuations likely by virtue of its large size compared with other habitats such as the wetland habitat (Bernardo & Melo, 2013). This high resilience might have caused the wooded grassland habitat maintain its habitat quality and quantity and thus its species composition throughout the year. Alternatively, it could be seasonal resource fluctuations in the other habitat types, rather than in the wetland habitat. For example, four of the seven species in the wetland habitat were recorded only during the dry season may be suggesting that some of the species may use the wetland habitat only during the dry season for various requirements such as water or forage which may be scarcely available in other habitats. Similar results have been demonstrated by a number of studies (Haugaasen & Peres, 2007; Alvarenga et al., 2018), suggesting that a combination of the wetland and the other habitats is crucial to the long‐term maintenance of viable populations of some species, such as the *K. ellipsiprymnus*.

In conclusion, findings of the study reveal that LANP supports a considerable number of medium and large mammalian species, including three globally threatened species: the endangered *L. pictus*, the vulnerable *P. pardus,* and *H. amphibius* (IUCN, 2020). This is the first ecological information on the diversity of mammals of the LANP, which would serve as a valuable baseline information for managers of the park to make effective conservation decisions and for researchers wishing to conduct related ecological studies. In addition to tightening law enforcement activities to reduce the current human and livestock encroachment into the park, studies on the population structure and spatiotemporal habitat use, and the impacts of human‐induced actions on the mammals of the park are needed to assist management plan formulation.

## CONFLICT OF INTEREST

The authors declare that there are no competing interests.

## AUTHOR CONTRIBUTIONS

**Guta Diriba:** Conceptualization (equal); methodology (equal); validation (equal); writing‐original draft (equal); writing‐review & editing (equal). **Sintaheyu Tamene:** Conceptualization (equal); investigation (equal); supervision (equal); writing‐review & editing (equal). **Girma Mengesha:** Conceptualization (equal); formal analysis (equal); writing‐original draft (equal). **Addisu Asefa:** Formal analysis (lead); methodology (equal); writing‐review & editing (equal).

## Supporting information

Appendix S1

## Data Availability

All data used are included in the article.
