# Supplementary material for: Diversity of medium and large mammals in the Loka Abaya National Park, southern Ethiopia
Source: Ecol Evol. 2020 Aug 28;10(18):9896–905. doi: 10.1002/ece3.6649 (PMC7520189; doi:10.1002/ece3.6649)
Supplement: Supplementary file 1 — Appendix S1 [file ECE3-10-9896-s001.docx]

Appendix 1. List of medium and large mammal species recorded in the Loka Abaya National Park and their relative abundance category (A = abundant; C = common; UC = uncommon; R = rare) in the four habitat types (WGL = wooded grassland; HS = hilly scrubland; RF = riverine forest; WL = wetland).

|  |  |  |  | **Relative abundance category** | | | |  |
| --- | --- | --- | --- | --- | --- | --- | --- | --- |
| **Order** | **Family** | **Common name** | **Scientific name** | **WGL** | **HS** | **RF** | **WL** | **All** |
| Carnivora | Canidae | Golden Jackal | *Canis aureus* | UC | R | R |  |  |
|  |  | Black-backed Jackal | *Canis mesamolas* | C | R | R |  |  |
|  |  | Bat-eared Fox | *Otocyon megalotis* | UC | R |  |  |  |
|  |  | African wild dog | *Lycaon pictus*^EN^ | C |  | R |  |  |
|  | Felidae | Serval Cat | *Felis serval* | R |  | R |  |  |
|  |  | Leopard | *Panthera pardus*^VU^ | R | R |  |  |  |
|  |  | African Wildcat | *Felis servestris* | R | R | R |  |  |
|  | Hyaenidae | Spotted Hyena | *Crocuta crocuta* | UC | R | UC |  |  |
|  | Vivarridea | Civet Cat | *Civettictis civetta* | R |  | R |  |  |
|  | Herpestidae | Egyptian Mongoose | *Herpestes ichneumon* | UC | R | R | UC |  |
| Artiodectyla | Bovidae | Greater Kudu | *Tragelaphus strepsiceros* | A | UC | A |  |  |
|  |  | Klipspringer | *Oreotagus oreotagus* | R | R |  |  |  |
|  |  | Common Bushbuck | *Tragelaphus scriptus* | C | R | UC | UC |  |
|  |  | Common Duiker | *Sylvicapra grimmia* | A | R | UC | R |  |
|  |  | Orib | *Ourebia ourebi* | UC | R |  |  |  |
|  |  | Lesser Kudu | *Tragelaphus imberbis*^NT^ | UC |  | R |  |  |
|  |  | Bohor Reedbuck | *Redunca redunca* | UC | R | R |  |  |
|  |  | Waterbuck | *Kobus ellipsiprymnus* | UC |  | R | C |  |
|  | Suidae | Common Warthog | *Phacochoerus africanus* | A | C | C | A |  |
|  |  | Bush Pig | *Potamochoerus larvatus* | C | R | R |  |  |
|  | Hippopotamidae | Hippopotamus | *Hippopothamus amphibius^VU^* | |  |  | C |  |
| Primates | Cercopithecidae | Anubis Baboon | *Papio anubis* | A | A | A |  |  |
|  |  | Grivet Monkey | *Chlorocebus aethiops* | A | R | A |  |  |
|  | Colobidae | Colobus Monkey | *Colobus geureza* | A |  | C |  |  |
| Logomorpha | Leporidae | Ethiopian Hare | *Lepus fagani* | C | R | UC |  |  |
| Tubulidentata | Orycteropodidae | Aardvark | *Orycteropus afer* | R |  | R |  |  |
| Rodentia | Hystricidae | Crested Porcupine | *Hystrix cristata* | R | R | R | R |  |
| Hyracoda | Procavidae | Rocky Hyrax | *Procavia capensis* | A | R |  |  |  |

Note:- For globally threatened species, their threat category is indicated in superscript letters following their scientific names, based on IUCN (2020), as: EN = Endangered, VU = Vulnerable, NT = Near Threatened.
